# Palliative care training addressed to hospital healthcare professionals by palliative care specialists: a mixed-method evaluation

**DOI:** 10.1186/s12904-019-0476-8

**Published:** 2019-10-26

**Authors:** Giovanna Artioli, Gabriele Bedini, Elisabetta Bertocchi, Luca Ghirotto, Silvio Cavuto, Massimo Costantini, Silvia Tanzi

**Affiliations:** 1 Palliative Care Unit, Azienda USL-IRCCS di Reggio Emilia, Viale Umberto I, 50, 42123 Reggio Emilia, Italy; 2 Casa Madonna dell’Uliveto, Centro Residenziale Cure Palliative – Hospice di Reggio Emilia, Reggio Emilia, Italy; 3 Qualitative Research Unit, Azienda USL - IRCCS di Reggio Emilia, Reggio Emilia, Italy; 4 Clinical Trials and Statistics Unit, Infrastructure Research and Statistic, Azienda USL - IRCCS di Reggio Emilia, Reggio Emilia, Italy; 5 Scientific Director, Azienda USL - IRCCS di Reggio Emilia, Reggio Emilia, Italy; 6 Clinical and Experimental Medicine, PhD program, University of Modena and Reggio Emilia, Modena, Italy

**Keywords:** Palliative care, Training evaluation, Health care professionals, Palliative care specialists, Hospital, Educational models

## Abstract

**Background:**

Despite the great advances in the concept of palliative care (PC) and its benefits, its application seems to be delayed, leaving unfulfilled the many needs of patients and family members. One way to overcome this difficulty could be to develop a new training programme by palliative care specialists to improve PC primary skills in healthcare professionals. The aim of this study was to evaluate the training’s impact on trainees within a hospital setting using Kirkpatrick’s and Moore’s models.

**Methods:**

We adopted a mixed-method evaluation with concurrent triangulation. The evaluation followed the first three steps of Kirkpatrick’s and Moore’s models and included a pre- and post-training evaluation through self-administered questionnaires and focus groups. We used the McNemar statistical test.

**Results:**

The results highlighted the significant amount of knowledge acquired by the hospital professionals after training, in terms of increasing their knowledge of palliative care and in terms of the change in meaning that they attributed to phenomena related to chronicity and incurability, which they encounter daily in their professional practice. In both quantitative and qualitative research, the results, in synthesis, highlight:

(i) the development of a new concept of palliative care, centred on the response to the holistic needs of people;

(ii) that palliative care can also be extended to non-oncological patients in advanced illness stages (our training was directed to Geriatrics and Nephrology/Dialysis professionals);

(iii) the empowerment and the increase in self-esteem that healthcare professionals gained, from learning about the logistical and structural organization of palliative care, to activate and implement PC;

(iv) the need to share personal aspects of their professional life (this result emerges only in qualitative research);

(v) the appreciation of cooperation and the joining of multiple competences towards a synergistic approach and enhanced outcomes.

**Conclusion:**

It is necessary to further develop rigorous research on training evaluation, at the most complex orders of the Kirkpatrick and Moore models, to measure primary PC skills in health care professionals. This will develop the effectiveness of the integration of I- and II-level palliative care competencies in hospitals and improve outcomes of patients’ and families’ quality of life.

## Background

Palliative care (PC) is a complex aspect of end-of-life (EoL) treatment, which focuses on providing compassionate medical care and preserving the patient’s dignity throughout the unfolding of a disease [1, 2]. Data from the literature largely confirm the effectiveness of PC [3–7] and encourage early access of patients to PC as well as the integration of PC into standard care [8–11]. Nonetheless, national- and international-level data [12] evidence the low referral rate of patients to PC [13]. This may most likely be due to sustainability issues, as PC requires ever more qualified PC specialists for a steadily growing patient supply and dwindling economic resources.

Moreover, the implementation of PC is not straightforward, as it implicates a broad range of aspects – from QoL to EoL interventions, from human relations to treatment costs [14–17]. Currently, the “modern” PC specialist is called on to cover multiple roles: being at the same time a clinician, a trainer (especially in the hospital setting) and a researcher with a natural attitude towards quality improvement [11]. However, it is unrealistic that PC experts meet all PC needs of incurable patients [14, 18].

One way to overcome such difficulties could be, as proposed by Quill and Abernethy [14], to structure PC on two levels: a I-level, so-called generalist PC, intended to meet the patient’s basic needs for PC in primary care and within hospital wards and involving transversal skills and knowledge common to all health professionals (HPs) in their daily clinical activity. The II-level is set up for more complex physical, psychological, social or spiritual needs and is carried out by specialized PC professionals [11]. The model, however, appears not to have been implemented so far, and there is still no indication of pursuing it in practical terms.

Some authors underline the importance of activating training courses to improve I-level skills among non-specialized PC professionals on basic principles of palliative care and to implement cooperation between the I and II levels [11, 14, 19].

Although the literature does describe several training interventions in PC [20–23], unfortunately, it rarely reports information about assessment tools used [24] or the methodological validity. A recent review by Turrillas et al. [25] has recognized an enormous lack of evidence for PC training evaluation.

In most cases, evaluation of training is mostly based on participant satisfaction and knowledge learning. A rigorous method is mandatory to evaluate the impact of a training course, according to the Medical Research Council framework (MRC) for complex intervention [26]. Nonetheless, the literature on this rigorous method is still underdeveloped [27, 28]. Accordingly, the aims of this study were to evaluate, both quantitatively and qualitatively, training’s impact on trainees while assessing the training’s model and evaluation method. For this study, we chosen to focus on hospital wards.

The study intends to make an important contribution by using a rigorous method for training evaluation in PC and to fill a gap in knowledge/education on PC for hospital healthcare professionals.

## Methods

We adopted a mixed-method evaluation with the concurrent triangulation, which consists of a qualitative and quantitative collection of data in the same period, in one subsequent separate analysis and, finally, in a comparison of the outcomes [29, 30].

We decided to carry out a before-after evaluation of the training, focusing on Moore [31] third order of learning, which is articulated in 3A (declarative knowledge) and 3B (procedural knowledge).

### The intervention

We developed a new training programme for PC based on the Kirkpatrick model [32, 33] and its elaboration by Moore [31]. The training programme, lasting 4 h in two different editions to facilitate participation, was taught by II-level PC specialists to HPs of all departments (I-level). The training focussed on the vision of PC by the World Health Organization (WHO) [1], the purpose of a PC unit in the hospital and the sharing of PC needs in hospital wards [7, 34].

### Context and sampling

The study was performed at the General Hospital “Arcispedale Santa Maria Nuova”, a 900-bed public hospital, located in Reggio Emilia, a town in northern Italy, recently awarded the title of Clinical Cancer Centre by the Organization of European Cancer Institutes (OECI). In the hospital, there is a Palliative Care Unit (PCU) assigned to provide specialist consultations for patients (and patients’ family members) both hospitalized in the hospital wards both afferent to the outpatient clinic. The PCU was established in April 2013 and currently includes three senior physicians, two advanced practice nurses and a specialist nurse in education.

The study sample was composed of HPs from the Radiotherapy, Geriatrics and Nephrology/Dialysis wards, who were involved in the basic PC training and who represented all professional categories implicated (physicians, nurses, technicians, biologists, etc.).

### Data collection and analysis

We used an open-ended questionnaire [35] about the comprehension of the WHO definition of PC, administered before and after training, asking: ‘Referring to your professional experience, what do you think are the goals and characteristics of PC?’. We analysed data using a framework constructed by Beccaro et al. [21], which is based on the 16 domains characterizing the WHO definition of PC. We calculated the percentages for each domain before and after training, along with the relevant 95% bilateral confidence intervals, constructed according to the Clopper-Pearson method. Pre- and post-training percentages were compared with the McNemar test; a *p*-value < 0.001 was considered statistically significant. The analysis was conducted using R3.3 (R Foundation for Statistical Computing, Vienna, Austria).

Qualitative feedback on PC knowledge and learning was gathered by means of focus groups (FGs) that served to encourage interaction between the participants, elicit a range of opinions/views and generate a discussion on the topic [36]. For each ward, we planned to conduct separate FG meetings for physicians and other HPs’ roles to bring out issues and topics characteristic of each professional profile. A moderator and an observer were present during every FG.

The qualitative analysis adopted the framework method described by Gale et al. [37]. We concentrated the analysis on emerging themes but also on emotions and meanings that the professionals attributed to their statements. In doing so, we could search for any possible changes in meanings attributed to that phenomenon from before to after the training. The overall process was supervised by an external expert of qualitative methodology.

We finally performed data triangulation to compare the quantitative and qualitative results [30].

## Results

The study included 80 HPs, accounting for 59.45% of the total staff of the 3 departments considered. Of these, 19 were physicians (Ph), 47 nurses (N) or technicians (T), and 14 other professional figures, such as nurse assistants and biologists. Of these, 33 participated in the pre-training FG and 29 in the post-training FG. Besides, we collected 77 pre-training and 77 post-training questionnaires as the same participants who filled both the questionnaires. We synthesized the training participants’ characteristics and their participation in the training course in Table 1.

**Table 1:** Training participants’ characteristics

Hospital ward	HPs	N	No. of participants	%
Nephrology Service and Dialysis	Physician	11	8	72.72%
	Nurse	50	18	36.00%
	Head Nurse	2	2	100%
	Nurse Assistant	6	6	100%
	Biologist	1	1	100%
	Total	70	35	50%
Geriatric Medicine	Physician	9	4	44.44%
	Nurses	16	12	75.00%
	Head Nurse	1	1	100%
	Nurse Assistant	15	4	35.71%
	Total	40	22	55.00%
Radiotherapy Service	Physician	8	7	87.50%
	Radiology Technician	16	7	43.75%
	Nurse	6	6	100%
	Head Nurse	2	1	50.00%
	Biologist	1	1	100%
	Nurse Assistant	2	1	50.00%
	Administrative Staff	2	/	/
	Total	37	23	59.45%
Grand total		147	80	54.42%

### Quantitative results

The open-ended questionnaire (before and after the intervention) gathered 77 different responses. Table 2 illustrates percentages, before and after, with confidence intervals and the outcome of the McNemar test [26] and the *p*-values for each domain.

**Table 2:** Distribution of WHO domains before and after training

Domain	WHO palliative care definition	Before	After	P
		Percentage with confidence interval	Percentage with confidence interval	*p*-value
D01	Improvement of patient quality of life	23.4 (14.5–34.4)	61.0 (49.2–72.0)	< 0.001
D02	Life-threatening illness	53.2 (41.5–64.7)	64.9 (53.2–75.5)	0.052
D03	Prevention and relief of suffering	32.5 (22.2–44.1)	28.6 (18.8–40.0)	0.689
D04	Treatment of pain	33.8 (23.4–45.4)	39.0 (28.0–50.8)	0.571
D05	Treatment of physical symptoms	22.1 (13.4–33.0)	40.3 (29.2–52.1)	0.001
D06	Psychological aspects of patient care	18.2 (10.3–28.6)	44.2 (32.8–55.9)	< 0.001
D07	Spiritual aspects of patient care	02.6 (00.3–09.1)	20.8 (12.4–31.5)	< 0.001
D08	Addressing patient and family needs	23.4 (14.5–34.4)	53.2 (41.5–64.7)	< 0.001
D09	Encouraging patients to live as actively as possible	01.3 (00.0–07.0)	09.1 (03.7–17.8)	0.077
D10	Helping family to cope during patient illness	10.4 (04.6–19.4)	23.4 (14.5–34.4)	0.016
D11	Helping family to cope with their bereavement	01.3 (00.0–07.0)	07.8 (02.9–16.2)	0.131
D12	Team approach in addressing needs	07.8 (02.9–16.2)	23.4 (14.5–34.4)	0.006
D13	Investigations aimed at improving management of clinical problems	00.0 (00.0–04.7)	00.0 (00.0–04.7)	/
D14	Early applicability in illness trajectory	03.9 (00.8–11.0)	23.4 (14.5–34.4)	< 0.001
D15	Affirming life	00.0 (00.0–04.7)	02.6 (00.3–09.1)	0.480

All 16 domains had entries among participants’ answers except for D13. The most significant difference was observed for domain D01 “Improvement of patient QoL”, which evidenced a statistically significant difference (*p*-value = < 0.001) between pre- and post-training, with an approximately three-fold increase.

Two other domains also showed significant enrichment with training: D14 “Early applicability in illness path” (*p*-value = < 0.001) and D08 “Addressing patient and family needs” (*p*-value = < 0.001).

The domains that concern addressing the patient’s holistic conception and taking charge of all its dimensions (D06 “Psychosocial aspects of patient care” and D07 “Spiritual aspects of patient care”) had significantly higher representation after the training intervention (*p*-value = < 0.001). These results also confirm that these domains had been properly learned during the course. These domains were followed in representation by D05 “Treatment of physical symptoms” (D05) (*p*-value = 0.001), which doubled after training; D12 “Team approach in addressing needs” (*p*-value = 0.006); and D10, referring to extending care to patients’ families, “Helping family to cope with their bereavement” (*p*-value = 0.016).

Finally, the D02 domain “Address the problems associated with incurable diseases”, with *p*-value = 0.052, and D09 “Encouraging patients to live as actively as possible”, had borderline statistical significance *p*-value = 0.077.

The significant differences between pre- and post-intervention were suggestive of changes in the understanding of the topics, increased awareness or acquisition of new notions.

### Qualitative findings

The analysis of the FGs before and after training led us to identify five overarching themes (Table 3): (1) Relationships among I- and II-levels, (2) Communication with patients and their families, (3) Clinicians’ competences in EoL care, (4) Integration among I- and II-levels, and (5) Self consideration of their emotions. These themes emerged with different meanings (defined within the sub-themes) in relation to pre-training and post-training data collection. We highlighted this meaning shift in Table 3.

**Table 3:** Meaning shifts among FGs from before training to after training

Themes		
Sub-themes emerging from FGs before the training	*← meaning shift →*	Sub-themes emerging from FGs after the training
‘Disagreement’	1. Relationships between I- and II-levels	‘Synergy’
‘Hard communication with patient and family’	2. Communication with patient and family	‘A collaborative approach to managing communication’
‘Perception of EoL care as useless’	3. Clinicians’ competences in EoL care	‘Becoming competent EoL care clinicians’
‘Initial perception that meeting and integrating with PC specialists is impossible’	4. Integration between I- and II-levels PC care	‘A possible integrative model with the PCU’
‘Difficulty in sustaining the emotional burden’	5. Mindfulness of their own emotions	‘Training course to support professionals’

#### Theme 1. Relationships between I- and II-levels, passing from obstacles to synergies

As the first result of the training, the meanings shifted from what we denominated “doubt and disagreement” to “knowledge and synergy”. Before the training, the HPs primarily emphasized a lack of specific guidance on PCU activities. They expressed the need to receive basic relevant information on PC, especially its objectives and its procedure, to advice a patient and her/his family about PC provision. The major deterrent was represented by the moment of the patient’s discharge from the hospital to hospice or home care. HPs revealed they needed information about how activate PC provision after hospitalization.

*“There needs to be clarity on the figures of reference for health professionals and PCU, the goals of PC, and how PCU works”* (FG 3 Ph).

*“One of the doubts I have is on when to call in palliative-care medical advice”* (FG 1 Ph).

Furthermore, there does not appear to be an official agreement indicating which hospital specialist is responsible for PC patient management. This not only creates obvious delays in requesting and providing assistance but also involves a conflicting decision-making process about treatments. Physicians and nurses, within the same wards, often disagreed on treatments. Participants were not fully aware of the local network, while they were aware of the weakness and the precariousness of their therapeutic programme, mainly when carried out at the patient’s home. HPs saw the risk that the continuity of care might disappear.

*“Our opinion was to suspend the treatment since the chances (of survival) were very few, but the specialist wanted to try a last line anyway, despite the patient … being already in desperate conditions. So there is no agreement between us”* (FG 1 Ph).

*“This is a problem that doctors often cause us … . They often demand things that are impossible to achieve”* (FG 2 N).

Once the hospital professionals received training on PC, they became aware of the broader picture and organization of PC, the paths for pursuing its integration within daily hospital activity and its implementation outside hospital structures. Additionally, having a better comprehension of the roles and profiles of the professionals working in this service allowed the other hospital professionals to be more attentive and sensitive in facilitating palliative doctor consultancies.

*“It was useful to get a clear definition of the meaning of palliative care, in relation to care and professional roles with them (PCU staff)”* (FG 7 Ph).

Having clarified that PC needs can actually be met by the PC specialist once a PC plan is activated led the participants to feel a sense of synergy with colleagues that seemed to facilitate inter-professional relations. The presence of the palliative specialist helped trainees to achieve a new perspective.

*“Now, after the training, there is a lot more awareness (on PC), and we can work in line with the medical staff, whereas before, when we had a patient in pain, we were in a situation where no one would take on the final provision for the treatment”* (FG 8 N).

*“With the introduction of the palliative doctor, we are facilitated in the assistance and decision making by coordination with the palliative doctor”* (FG 11 Ph).

#### Theme 2. Communication with patient and family: ‘a collaborative approach to manage communication’

The relationship and communication with the patient’s family were reported as particularly strenuous by the professionals. Among the aspects highlighted by the participants were dealing with suffering and incurable conditions, the nature of the information they are in charge of communicating (e.g., poor prognosis, recurrent disease, last line of active treatment, transition from active care to PC) and the underlying issues of communication skills and personal ethics.

“*I'm faced with the dilemma*: “*What* should we communicate and *how*?” “*I sometimes ask myself whether I should communicate the diagnosis or not*” (FG 7 Ph).

“*Since I do not know what the patient's wishes are, I often refer to the family's judgement*” (FG 7 Ph).

“*Each of us is self-taught in this field, but in reality, there are communication techniques that we absolutely do not know; we lack communication training*” (FG 1 Ph).

Participants felt that communication with the family was very problematic and listed some typical situations, such as when the family members cannot accept the fact their relative’s disease is inevitably worsening towards death or when a family member asks to continue a useless therapy.

“*It is necessary to help the family understand the meaning of pain therapy in relation to the process of accompanying the patient towards death; many times, the medical staff is forced to resort to a defensive medicine, just because the family are not entirely convinced that this person, in the end, is dying*” (FG 5 Ph).

They stressed that this issue remained a critical point despite having undergone the present training course and began planning further training that could help them address their communication needs towards a collaborative approach with PC professionals.

“*It is important to organize the approach together with colleagues, with the patient and his/her family, a possible caregiver*” (FG 1 Ph).

“*Our problems with communication lie largely in the lack of training*” (FG 3 Ph).

#### Theme 3. Clinicians’ competences in EoL care: ‘becoming competent clinicians of the EoL’

Participants shifted from what we called “sense of uselessness towards the EoL” to “discovering the ‘treatments’ of the EoL”. In fact, participants felt a sense of uselessness, of ‘having their hands tied’. Before the training, pain was a cause of conflict among nurses and physicians: on one hand, the physicians considered the problem of the patient’s pain already solved, while the nurses thought there was a lack of pain therapy culture. Participants were more sensitive to the patient’s global suffering:

*“The problem is precisely the therapy of pain, which perhaps we have not yet understood how it should be done, we see the patient suffering, but sometimes we have our hands tied”* (FG 4 N).

*“We often ask family members to allow us to give (*PC) *a try, … for something that will probably prolong the patient's suffering and that brings no apparent advantage to the patient's well-being. So, there is an insecurity that we all have towards these choices”* (FG 1 Ph).

The participants discovered the importance of EoL treatments, as they reported a higher sensitivity and attention to the patient at the EoL after training. Nurses recognized that physicians, after the training, were more likely to involve palliative doctors, when before they felt more embarrassed. All the HPs gained a clear understanding of the area of intervention of PC, namely, the response to the needs of the person, even when they could no longer manage the treatment of the disease.

Nevertheless, the problem of identifying psychosocial and spiritual needs remained in both the pre- and post-training FGs.

Participants understood that the intervention of the PC specialist is needed to address the complexity and multiplicity of symptoms and needs that cause suffering in the patient.

*“Surely our doctors have become confident enough to call the palliative doctor, and this alone is an important step ahead”* (FG 10 N).

*“For our work, we should have a greater knowledge of what palliative care is”* (FG 9 Ph).

#### Theme 4. Integration between I- and II-levels: ‘a possible integrative model with the PC Unit’

From the “initial perception of the impossibility of meeting and integrating the PC specialists” and being somewhat powerless, participants shifted to an “understanding of the process, which will facilitate PC inclusion in their work”. In fact, the first reaction of the professionals revealed their doubts about the feasibility of PC. The expectations of participants before the training were to receive not only theoretic notions but also concrete proposals they could transfer to the patient’s bedside. The first obstacle was traced back to organizational problems. HPs declared that working schedules were short and that the organization often did not allow a method that was different from the one traditionally intended, where it is the doctor alone who interacts with the patient and the family. Participants thought that more complex approaches, such as those proposed by palliative specialists involving several actors, were challenging to apply.

*“I am often involved in the most difficult part of setting up the PC programme and coordinating everyone, from my colleagues to the patient, family, and a possible caregiver”* (FG 1 Ph).

*“Even doctors, however, are always struggling to involve the palliative doctors. They always wait until the last moment”* (FG 4 N).

The participants’ understanding of the organization within the PCU seemed to have facilitated the involvement of the palliative doctor’s consultancies within the care. Participants became aware of the practical possibility of activating PC and showed the need for greater integration of PC specialists within the examined operating units (OUs).

*“We should involve palliative specialists more and more, so they can get to know our patients, the dynamics at our department, and grasp the mesh of our organization”* (FG 9 Ph).

Still, some HPs perceived organizational boundaries and constraints.

*“What we will discover is whether we can implement PC on a practical basis, with the patient. However, in this setting, there is neither the time nor the mindset to use this approach to the patient. Because we are unable to do it... because of the organization”* (FG 3-4 N & Ph).

#### Theme 5. Self-consideration of their emotions: ‘training course to support professionals’

The last theme emerging from FGs regarded the difficulty of the professionals in sustaining, over time, the emotional charge of daily contact with patients and families who have EoL situations.

Following training, professionals in the FGs recognized above all the need to receive psychological support themselves to face complex EoL situations.

*“Plus, we don't receive much attention ourselves. In fact, the staff suffer... from an emotional point of view. You deal with it on your own. You can take an individual route, but it's not enough. For years we've been claiming that we need support for the staff”* (FG 2 N).

The risk of burden was perceived especially by nurses, who felt they would gradually become unable to help anymore because of the intensity of emotions experienced. HPs reported that they were not able to identify, welcome and process those strong feelings. Both personal development and preparedness were needed to cope with the phenomenon.

*“Manage this impact requires the right maturity, and the right preparation; the psychological aspect is important for the family, it is important for the doctor, but it is also important for the nurse and the assistants who are in contact with the patient on a daily basis”* (FG 2-6 N & T).

From the post-training FG, the previously highlighted needs and the fears expressed by the participants related to the risks of emotional stress led the way to a precise awareness. It appeared that the requests for help became explicit, even if not yet well circumstantiated: from training to communication to the care for HPs, to emotional support in the management of complex cases. Participants recognized the training as an occasion for discussion and sharing of the most complex problems. The shared search for common solutions could become a support strategy.

*“We should establish a mutual-support group. Moments to dedicate not only to the discussion of cases but also of what has caused emotional reactions in the colleagues”* (FG 8 -12 N & T).

### Triangulation outcomes

The participants were very active and participatory in the whole path of training and research, allowing the collection of very interesting data in both our quantitative and our qualitative research. The triangulation of data led, in most cases, to both confirmatory and novel results.

The results obtained highlighted the significant amount of knowledge acquired by the participants after training, in terms of increasing the knowledge on PC and in terms of the change in meaning that they attributed to phenomena related to chronicity and incurability, which they encounter daily in their professional practice.

In both quantitative and qualitative research, the results, in synthesis, highlight:

- the development of a new concept of PC, centred on the response to the holistic needs of people (Domains: D06, D07, D08 and Theme 3);
- the understanding that PC can also be extended to non-oncological patients in advanced illness stages (our training was direct to Geriatrics and Nephrology/Dialysis professionals);
- the empowerment and increased self-esteem that HPs gained, from learning about the logistical and structural organization of PC, to activate and implement PC (Domains: D01, D014, Theme 1, 3 and 4);
- the need to share personal aspects of their professional life (this result emerged only in the qualitative research: Theme 5);
- the appreciation of cooperation and the joining of multiple competences towards a synergistic approach and enhanced outcomes (Domain: D012 and Theme 4).

## Discussion

The present work describes the piloting of a new training programme along with its evaluation.

This study evaluates a basic PC training programme in a hospital by means of a fittingly rigorous methodology, in view of implementing the II-level PC model dealing with patients who are candidates for PC.

The adoption of the validated models of Kirkpatrick and Moore and a mixed-method approach allowed us to explore the phenomenon of PC as a whole. In particular, through quantitative data, we evaluated the increase in awareness, while the emerged meaning shift revealed both what the participants learned and how they signified what they learned.

In agreement with Schenker and Arnold [18, 38, 39], to improve PC for patients with chronic illness, we used specific training to develop I-level PC skills in HPs to improve the quality of care in hospital. Our results are in agreement with other studies that were also applied in non-oncological settings, for example, those obtained by Hepgul et al. [7], which emphasized that the collaboration between neurologists and palliative care professionals has a positive overall impact on the management of patients with progressive neurological disorders. Similar results were obtained by Bowman and Meier [40] in chronic obstructive pulmonary disease and Riegel and Kimmel [41] in end-stage heart disease.

The results obtained highlighted the significant amount of knowledge acquired by the participants after training, in terms of increasing their knowledge of PC and in terms of the change in meaning that they attributed to phenomena related to chronicity and incurability, which they encounter daily in their professional practice [42]. Turrillas et al. [25], through a systematic review of the existing literature on the subject, showed that in the analysed studies, despite the use of various original or modified educational training and assessment methods of which psychometric characteristics were often not reported, all educational methods have allowed non-palliative care professionals to improve their knowledge and preparation regarding palliative care and EoL patient management.

In our study, the qualitative analysis showed that meaning shifts emerged both as an interesting result of the training and as an innovative proposal for training evaluation.

Our training course has helped the HPs (i) develop a new concept of PC centred on the response to the needs of people [43] and not only to care; (ii) understand that PC can also be extended to non-oncological patients [44, 45] in advanced illness stages; (iii) gain empowerment and increased self-esteem from learning about the logistical and structural organization of PC to activate and implement PC [46–48]; (iv) appreciate the need to share personal aspects of their professional life; (v) appreciate the cooperation and joint multiple competences that enable a synergistic approach and enhanced outcomes [7, 49].

Both HPs and PC specialists mentioned throughout the course their need for multidisciplinary team support to improve care processes; the need to work together emerged, as did the need to share choices and to promote integration among the various kinds of professionals [50].

These unmet needs, which all fall under the umbrella of PC in the hospital setting, were eventually summarized in the request for immediate development of additional training modules, one for each specific topic/unmet need, such as management of personal emotions [51], communication skills for delivering bad news to patients and their families [52], and holistic patient management [53]. In fact, after this study, participants requested that training boards organize a course that could deepen their knowledge on psycho-social needs and advanced training in bad-news communication for physicians, while the PC Unit has recently begun a new qualitative study on spiritual needs in chronic illness patients.

This study has some limitations. The number of questionnaires collected was not high, although the statistical analysis allowed us to identify significant differences. We measured only the impact on professional competencies and not effectiveness on patients and health outcomes.

## Conclusions

There is a growing need to implement new PC training delivery models [54]. We wanted to experiment with the model in which PC specialists (II-level) help hospital clinicians develop I-level skills through a training course, and the results were rigorously measured.

This study intended to propose a training evaluation method that used both quantitative and qualitative data; this method could also be applied to other training courses. However, it will be necessary to go beyond the learning of applicable knowledge and new meanings to propose training that develops measurable skills in professionals, as well as outcomes to improve patients’ and families’ QoL. It is necessary to conduct further rigorous research on the training evaluation, at the most complex orders of the Kirkpatrick and Moore models, to measure the effectiveness of the integration of I- and II-level PC competencies in care pathways and to evaluate patient- and family-related outcomes.

## Data Availability

The study documentation is collected and managed by the coordinator of the study centre (PC Unit, AUSL – IRCCS di Reggio Emilia), and datasets are available on reasonable request.

## Abbreviations

EoL: End-of-life
FG: Focus group
HP: Healthcare professional
MRC: Medical Research Council
N: Nurse
OECI: Organization of European Cancer Institutes
OU: Operating unit
PC: Palliative care
PCU: Palliative Care Unit
Ph: Physician
QoL: Quality of Life
T: Technician
WHO: World Health Organization
